# The Effect of Using Peer on Self-Care, Quality of Life, and Adherence in Elderly People with Coronary Artery Disease

**DOI:** 10.1155/2021/4770721

**Published:** 2021-11-11

**Authors:** Mojtaba Roshandel, Mahnaz Rakhshan, Majid Najafi Kalyani

**Affiliations:** School of Nursing and Midwifery, Shiraz University of Medical Sciences, Shiraz, Iran

## Abstract

**Introduction:**

Coronary artery disease is one of the most common diseases and the cause of death among elderly people. Due to the chronic nature of this disease, regular follow-up, lifestyle changes, and adherence to recommendations can reduce the complications and improve the quality of life among elderly individuals. Given the importance of using educational methods that are based on the patient's age and disease stage, the present study aimed to investigate the effect of using a peer group on self-care, adherence, and quality of life in elderly people.

**Method:**

This single-blind clinical trial was conducted on 30 old patients with coronary artery disease aged 60 years in Shiraz from March to June 2021. The patients were selected using simple random sampling and were then randomly assigned to the peer education and control groups (15 participants in each group) via permuted block randomization. The patients in the peer education group received the necessary education about medications, lifestyle, daily activities, self-care, and adherence through three educational clips by educated peer. The patients in the control group received routine education using two clips by the ward nurses. The levels of self-care, adherence, and quality of life were assessed in the intervention and control groups before and one month after the intervention. Data analysis was performed by SPSS 20 software using an independent *t*-test, paired sample *t*-test, and chi-square test. *P* < 0.05 was considered statistically significant. *Findings*. The results showed no statistically significant difference between the two groups with respect to the mean scores of self-care and quality of life before the intervention (*P* > 0.05). Following the educational intervention, however, a statistically significant difference was found between the two groups concerning the mean scores of self-care and adherence (*P* < 0.05). Moreover, the mean differences between the two groups regarding the three variables were statistically significant before and after the intervention (*P* < 0.05).

**Conclusion:**

Education based on multimedia clips by peer was effective in increasing the mean scores of self-care, adherence, and quality of life among the elderly people with coronary artery disease. Given the low cost, high effectiveness, and decrease in the nurses' workload, this method is recommended to be used alongside other methods in order to educate elderly individuals suffering from coronary artery disease.

## 1. Introduction

Old age is one of the important periods of life experienced by all people [1]. The increasing growth of the elderly population has been referred to as the silent revolution [2]. Elderliness is among the important social and health challenges of the 21^st^ century, and according to predictions, the population of individuals over the age of 60 years will reach more than two billion by 2050 [3, 4]. In the elderliness period, the prevalence of diseases follows an ascending trend. Cardiovascular diseases are among these diseases, which are increasing rapidly in developing countries [5]. According to the assessments carried out by the World Health Organization (WHO), approximately 23.6 million people will die because of cardiovascular diseases by 2030 [6]. In Iran, cardiovascular diseases are the most common cause of death [7]. According to the latest report by the Ministry of Health, around 300 people in Iran die due to cardiovascular diseases every day [8]. Coronary artery disease is one of the most common cardiovascular diseases among elderly people, which occurs with the progress of such cardiovascular lesions as coronary artery stenosis and obstruction. They are the main cause of death among elderly individuals [9]. Given the chronic nature of this disease, follow-up and adherence to care and treatment guidelines including self-care behaviors and adherence to treatment form one of the factors determining and affecting complications and preventing the exacerbation of symptoms in these patients [10, 11].

The concept of “self-care” was originally introduced by Orem. It refers to the learned behaviors that people show to maintain or improve their life, health, well-being, prevention, and treatment [12, 13]. Adherence to treatment is another important pillar of patient care programs. Accordingly, patients should adapt their behaviors to the recommendations provided by healthcare providers to ensure the effectiveness of the treatment regimen for their disease [10]. Adherence to self-care behaviors and adherence to treatment not only prevent the exacerbation of the signs and symptoms but also reduce the hospitalization frequency, increase well-being, reduce treatment costs, and increase the quality of life of patients with chronic diseases [13, 14]. One of the main issues in improving the life quality of elderly people is maintaining their independence in physical and cognitive activities and living actively and independently [15].

There are different methods for improving the quality of life, self-care, and adherence amongst patients with chronic diseases [13, 16]. Patient education is one of such effective methods and strategies [17]. One of the effective methods in improving patient education, especially in the elderly group, is using the knowledgeable members of the society who are called nonspecialist health advisors or peer educators [18]. A peer is a person who belongs to the same social group, and people believe that he/she is similar to them with regard to their abilities and can have strong and positive effects on motivation and improvement of learning [19]. Education by peers is one of the educational approaches that aim to develop knowledge and attitude and create healthy behaviors through individuals who are not professionally trained but have common experiences. In fact, peer education is a novel educational approach that includes identification, education, and support of the members of a group for the transfer and communication of accurate information to others with similar characteristics and properties [17]. Methods used for peer education include workshops, group discussions, and one-on-one discussion, and different methods have to be used depending on different situations [20]. In peer education, since the peer and the patient are the members of the same group, the sense of empathy and social identity are strengthened, leading to an increase in knowledge and awareness [21]. Due to the similar nature of the disease, common experiences, and similar feelings, patients accept their peers' information more easily and share their beliefs and feelings with them [22, 23]. Peer education can be implemented in different environments and various situations. In the past decades, peer education was mostly used to prevent addiction, drinking, and drug abuse. In the recent years, this educational approach has been used in a wider range of cases such as arthritis, anxiety, cardiac diseases, AIDS, breast cancer, burns, and diabetes [20].

Considering the high prevalence of coronary artery disease among elderly individuals, importance of educating these patients for improving self-care, quality of life, and adherence, shortage of nurses in hospitals, advantages of education using peer groups, and lack of adequate studies on this subject matter, the present study aims to assess the effect of educational multimedia clips using a peer group on self-care, quality of life, and adherence among elderly individuals with coronary artery disease.

## 2. Method

### 2.1. Design

This single-blind clinical trial was conducted in Shiraz from March to June 2021 in order to determine the effectiveness of peer education in the elderly people suffering from coronary artery disease.

### 2.2. Participants

In this study, 30 patients were selected using simple random sampling after assessment of the inclusion and exclusion criteria. The inclusion criteria of the study were aging between 60 and 85 years, not suffering from any known psychological diseases, not using psychiatric medications based on the patients' statements, having coronary artery disease, not having any cognitive, speech, or auditory impairments, not having pain during education, being willing to cooperate, being able to practice self-care and adhere to treatment, having full consent to participate in the study, and completing the informed consent form. According to the study conducted by Golaghaie et al. [17] and considering the total scores of self-care in the intervention and control groups, *a* = 0.05, and power = 80%, a 24-subject sample size was estimated (12 participants per group). To increase the study power and reduce the sample attrition risk, the sample size was increased by 20%, and finally, 15 patients were considered for each group (a total of 30 patients).

### 2.3. Randomization

**Figure 1:**
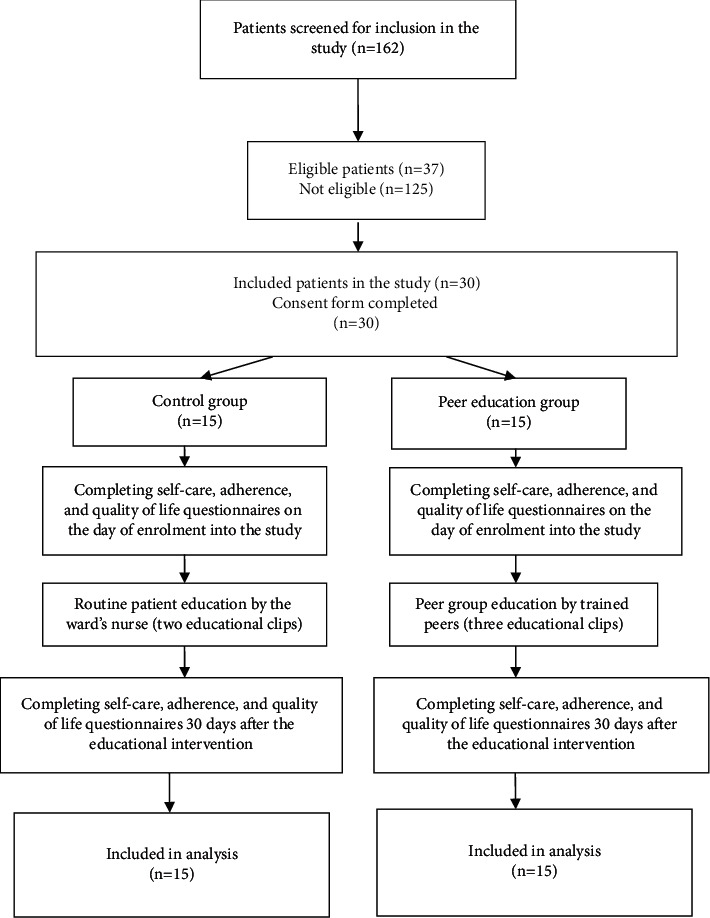
Different steps of the study based on the CONSORT guidelines.

After fully explaining the study method to the patients and obtaining their written informed consent forms, the patients were randomly assigned to the peer education and control groups using permuted block randomization. The patients were assigned to the groups by flipping a coin on a weekly basis in order to prevent contact between the patients in the two groups. As a result, the patients in the study groups could not interact with each other. The steps of conducting the study have been presented in Figure 1.

### 2.4. Intervention

The patients in the peer education group received three educational clips (nearly 20 minutes each) about coronary artery disease provided by the peer. The educational content included drug regimen (the prescribed drugs and diet (food matching the disease conditions)) and patients' activities (how to perform daily activities). Education was provided by the peer for the patients using three multimedia clips sent via WhatsApp. The peer was selected based on the following criteria: being a volunteer, having at least a high school diploma, having been under treatment for at least six months, proper articulation, proper communication, being interested in education, being educable, and being willing to educate the patients. Education of the peer was carried out by a researcher via lecture, question and answer, and group discussion methods through two 60-minute sessions as well as by role-play at home. To ensure the reception of education by the peer, the items were discussed at the end of the educational sessions. The patients in the control group received the routine ward care and education by two educational clips provided by a CCU nurse. The education was carried out via two clips (almost 15 minutes) about the disease, drug regimen (prescribed drugs and diet (food suiting the disease conditions)), and patients' activities (how to perform daily activities).

### 2.5. Measurement

In this study, after assigning the patients to the peer education and control groups, the demographic information questionnaire, the 34-item self-care scale, the 40-item adherence questionnaire, and the 12-item quality of life questionnaire were completed by the patients (pretest). One month following the educational intervention, the patients were asked to complete the 34-item self-care questionnaire, the 40-item adherence questionnaire, and the 12-item quality of life questionnaire via telephone contact (posttest). The 34-item self-care questionnaire, the 40-item adherence questionnaire, and the 12-item quality of life questionnaire have been used in numerous studies, and their validity and reliability have been confirmed [24–26]. The self-care questionnaire consisted of 34 questions designed in four dimensions, namely, physical self-care (ten questions), psychological self-care (six questions), emotional self-care (nine questions), and spiritual self-care (nine questions). The minimum and maximum scores of this questionnaire were 34 and 170, respectively. The validity and reliability of this questionnaire were confirmed with the correlation coefficient of 0.83 in a previous study [27].

The 12-item quality of life questionnaire was the truncated form of the 36-item quality of life questionnaire, which has been widely used in various studies. This questionnaire contained eight subscales. Considering the small number of items, the overall scores of the respondents were used. The questionnaire evaluated the quality of life with regard to the overall understanding of one's health, physical performance, physical health, emotional problems, physical pain, social performance, joy and vitality, and mental health. The validity and reliability of this scale were assessed in the study conducted by Montazeri et al. in Iran and were confirmed with Cronbach's alpha coefficients of 0.72 and 0.73 for the mental dimension and the physical dimension, respectively [28].

The adherence questionnaire for chronic patients was designed and subjected to psychometric analysis by Modanlou in 2013. This questionnaire consisted of 40 questions about adherence (nine questions), willingness to participate in the treatment (seven questions), adaptability (seven questions), integration of treatment with life (five questions), complete adherence to treatment (four questions), commitment to treatment (five questions), and wise implementation of the treatment (three questions). The questionnaire items could be responded via a five-point Likert scale ranging from fully agree to fully disagree (scores five to one). Accordingly, a score between 75% and 100% showed excellent adherence to treatment, a score between 74% and 50% represented good adherence to treatment, a score between 26% and 49% indicated average adherence to treatment, and a score between 0% and 25% showed poor adherence. The validity and reliability of this scale were confirmed in Modanlou's study with a correlation coefficient of 0.875 (*r* = 0.875) [24].

### 2.6. Ethical Considerations

This study was conducted after obtaining permission from the Ethics Committee of Shiraz University of Medical Sciences (IR.SUMS.REC.1398.524). After explaining the study goal and methodology, written consent forms were obtained from the patients. Besides, the patients were assured that their information would be kept confidential and that they could stop participating in the study at any stage.

### 2.7. Statistical Analysis

After collecting and encoding the information, data analysis was performed by SPSS software, version 20. Chi-square and independent *t*-tests were used to examine the two groups regarding the demographic variables. Besides, an independent *t*-test and paired sample *t*-test were utilized to compare the two groups regarding the quality of life, self-care, and adherence. *P* < 0.05 was considered statistically significant.

## 3. Results

All patients participating in this study including 30 patients in the intervention and control groups entered the final phase of analysis. According to Table 1, there was no statistically significant difference between the two groups with regard to age, gender, marital status, education level, profession, and history of hospitalization.

Before the intervention, the results showed no statistically significant difference between the two groups with respect to the mean scores of self-care and quality of life (*P* > 0.05) (Table 2).

The results of the paired sample *t*-test showed a significant difference in the peer education group's mean scores of self-care, adherence, and quality of life before and after the intervention (*P* < 0.05). In this group, the mean scores of self-care, adherence, and quality of life increased after the intervention compared to the baseline. In the control group, the mean scores of self-care and adherence increased after the intervention compared to the baseline.

According to Table 3, a significant difference was observed between the two groups regarding the mean scores of self-care, adherence, and quality of life (*P* < 0.05). Furthermore, the differences between the mean scores of the three variables before and after the intervention were significantly higher in the intervention group compared to the control group (Table 3).

## 4. Discussion

The present study results demonstrated that the use of multimedia-based peer education led to a significant increase in the mean scores of self-care, adherence, and quality of life among the elderly individuals with coronary artery disease compared to the control group. The results of the study conducted by Ghasemi et al. were in line with those of the present study, indicating that the use of a peer group increased the self-care scores of the elderly people suffering from diabetes [25]. Similarly, Ahmadi et al. showed in their study that the use of peer education increased the self-care scores of the elderly patients suffering from diabetes [26]. In another study carried out by Shojafard et al., the use of peer education increased the self-care scores of the patients suffering from heart failure [29]. The results of the study conducted by Heisler et al. titled “Peer Support for Self-Care in Patients with Diabetes” also revealed that peer education led to an increase in self-care, the correct use of medications, and a decrease in the need for insulin in the peer group compared to the control group [30]. Chaffey and Bigby too carried out a study and reported that the use of peers improved the knowledge of health management and increased self-care in patients with spinal cord injury [31]. Moreover, diabetes self-care education by peer groups increased the self-care behaviors of these patients in a study performed by Gatlin et al. [32]. Consistently, the findings of the research by Hasanah et al. showed that education using peer groups increased adherence in patients with pulmonary tuberculosis [33]. Furthermore, Khavasi et al. disclosed that peer education was a useful and effective method of increasing self-efficacy in patients with diabetes and could enhance their ability to adhere to the treatment regimen [34]. Overall, peer education was highly effective and reduced the cost of educating the elderly people because peers could communicate more effectively, influence their peers, and encourage them to adhere to the treatment [35]. The results of the study performed by Sadeghi et al. on elderly individuals with hypertension indicated that the use of a peer group could be an effective measure for increasing adherence to diet and improving blood pressure control amongst elderly people [36]. In line with the present study, the results of all aforementioned investigations indicated that peer education increased self-care and adherence scores in the elderly individuals suffering from chronic diseases.

The results of the current study demonstrated that the use of a peer group for educating the elderly people increased their quality of life scores. The results of the study conducted by Daryadokht et al. also indicated that peer education improved the quality of life in patients with hypertension [37]. In the same line, the results of the study conducted by Mohsenikhah et al. revealed that the use of a peer group increased self-care and quality of life in patients with diabetes [38]. In addition, Golaghaie et al. reported that the use of a peer group as a patient education method promoted the quality of life in patients [17]. In another research carried out by Jahanshahi et al., peer education led to an improvement in the quality of life of patients with heart failure [39]. Moreover, Borzou et al. compared the effects of individualized and peer education methods on the quality of life of patients with heart failure and concluded that both education methods increased the quality of life, but peer education had a greater effect in the long run [35]. Similarly, the results of the study conducted by Sharif et al. indicated that peer education influenced the quality of life of mastectomy cases and that the mean score of quality of life increased compared to before the intervention [40]. In contrast, the results of the study conducted by Rashidi et al. showed no changes in the blood sugar levels and intake of diabetes medications after three months of peer education about diabetes self-care [41]. Azizi et al. also conducted a study on the effect of education about HIV/AIDS prevention by peers, physicians, and pamphlets on the awareness of female high school students and stated that peer education did not increase awareness in the intervention group [42]. Moreover, the results of the study conducted by Morowati et al. suggested that peer education was not effective in increasing the level of awareness [43]. According to Simmons et al., the factors influencing peer education on self-care included the selection method, education style for educating peers, participants' knowledge level about diabetes, and other factors [44]. Yet, the results of the study performed by Ebrahimi et al. showed that educational intervention using mobile phones was effective in women's lifestyle since a large population in a wide geographical area could be educated [45].

One of the most important strengths of this study was the simultaneous analysis of self-care, adherence, and quality of life among the elderly individuals with coronary artery disease, which was not addressed in the previous studies. Although the patients in the two groups did not have any interactions with each other, the acquisition of information by the patients through other sources was not controlled, which could be a potential limitation. Another study limitation was its small sample size due to the COVID-19 pandemic and selection of patients from one hospital. Thus, future studies are recommended to be conducted on a large number of patients selected from different hospitals. Another study limitation was the short interval between the intervention and assessment of the patients' outcomes. Since patients' outcomes may change after recovery and returning to routine daily activities, further studies are suggested to consider longer time periods for assessing patients' outcomes. The final study limitation was the utilization of self-report scales for analyzing the patients' outcomes.

## 5. Conclusion

The findings of the present study indicated that the use of multimedia-based peer education increased the scores of self-care, adherence, and quality of life in the elderly individuals suffering from coronary artery disease. Given that this method is a low-cost one, it is recommended to be used by healthcare providers alongside other education methods in order to educate elderly people with coronary artery disease.

## Figures and Tables

**Table 1:** Characteristics of the participants.

	Peer education (*n* = 15)	Peer education (*n* = 15)	Control (*n* = 15)	*P* value^*∗*^
		Frequency (percent)	Frequency (percent)	
Gender	Male	9 (60)	8 (53.3)	0.713
	Female	6 (40)	7 (46.7)	
Marital status	Married	15 (100)	15 (100)	1.0
	Widowed	0	0	
History of hospitalization	Yes	10 (66.7)	15 (100)	0.014
	No	5 (33.3)	0 (0)	

^*∗*^Chi-square test.

**Table 2:** Comparison of the two groups regarding the mean scores of self-care, adherence, and quality of life.

Group variable	Before the intervention		
	Mean ± SD		
	Self-care	Adherence	Quality of life
Peer education	112.86 ± 5.96	68.80 ± 4.090	27.60 ± 4.65
Control	116.20 ± 7.25	72.06 ± 3.01	30.0 ± 6.16
*P* value^*∗*^	0.118	0.019	0.239

^*∗*^Independent sample *t*-test.

**Table 3:** Comparison of the mean differences of self-care, adherence, and quality of life scores before and after the intervention.

Group variable	Peer education	Control	Mean difference	95% confidence interval		*P* value^*∗*^
	Mean ± SD	Mean ± SD	Mean ± SD	Lower	Upper	
Self-care	18.46 ± 7.93	7.06 ± 3.71	11.40	6.76	16.03	<0.001
Adherence	10.93 ± 4.75	3.83 ± 1.55	7.10	4.45	9.74	<0.001
Quality of life	8.20 ± 4.69	2.73 ± 1.62	5.46	2.83	8.09	<0.001

^*∗*^Independent sample *t*-test.

## Data Availability

The data used to support the findings of this study are available from the corresponding author upon reasonable request.
